# Supplementary material for: Bleaching causes loss of disease resistance within the threatened coral species Acropora cervicornis
Source: eLife. 2018 Sep 11;7:e35066. doi: 10.7554/eLife.35066 (PMC6133546; doi:10.7554/eLife.35066)
Supplement: Supplementary file 1. [file elife-35066-supp1.docx]

Supplementary file 1. Results of the two-sample t-tests and Kruskal Wallis tests comparing the photochemical yield of 15 different genotypes of *Acropora cervicornis* prior to bleaching in August 2015 and after bleaching occurred in September 2015.

| Genotype | t-value | Degrees of Freedom | p-value |
| --- | --- | --- | --- |
| 1 | -10.13 | 22 | p<0.001 |
| 3 | -9.82 | 22 | p<0.001 |
| 5 | -10.80 | 22 | p<0.001 |
| 10 | -8.90 | 22 | p<0.001 |
| 13 | -10.55 | 22 | p<0.001 |
| 41 | -8.15 | 18 | p<0.001 |
| 44 | -7.33 | 18 | p<0.001 |
| 46 | -8.43 | 23 | p<0.001 |
| 47 | -6.60 | 18 | p<0.001 |
| 57 | -7.94 | 19 | p<0.001 |
| 58 | -8.28 | 18 | p<0.001 |
|  |  |  |  |
| Genotype | X^2^ | Degrees of Freedom | p-value |
| 4 | 17.16 | 1 | p<0.001 |
| 7 | 17.16 | 1 | p<0.001 |
| 9 | 18.14 | 1 | p<0.001 |
| 50 | 14.33 | 1 | p<0.001 |
