# Supplementary material for: Bleaching causes loss of disease resistance within the threatened coral species Acropora cervicornis
Source: eLife. 2018 Sep 11;7:e35066. doi: 10.7554/eLife.35066 (PMC6133546; doi:10.7554/eLife.35066)
Supplement: Supplementary file 2. — Replicate samples of the same host genet contained the same S. fitti genotype in all cases. Host genets 3, 4, 5, 7, 9, 10, 13, 41, 44, 47 and 58 harbor S. fitti strain F421. [file elife-35066-supp2.docx]

Supplementary file 2. Table S2: *Symbiodinium fitti* multilocus genotypes. Replicate samples of the same host genet contained the same *S. fitti* genotype in all cases. Host genets 3, 4, 5, 7, 9, 10, 13, 41, 44, 47 and 58 harbor *S. fitti* strain F421.

| **Field ID** | **Locus (PCR product length in bp)** | | | | | | | | | | | | | **Symbiont**  **Clonal ID** |
| --- | --- | --- | --- | --- | --- | --- | --- | --- | --- | --- | --- | --- | --- | --- |
|  | **1** | **3** | **7** | **9** | **18** | **27** | **28** | **31** | **32** | **41** | **2** | **8** | **48** |  |
| 1 | 214 | 189 | 177 | 135 | 209 | 196 | 226 | 215 | 248 | 135 | 179 | 129 | 175 | F419 |
| 1 | 214 | 189 | 177 | 135 | 209 | 196 | 226 | 215 | 248 | 135 | 179 | 129 | 175 | F419 |
| 3 | 212 | 189 | 177 | 135 | 209 | 196 | 226 | 215 | 248 | 135 | 179 | 129 | 175 | F421 |
| 3 | 212 | 189 | 177 | 135 | 209 | 196 | 226 | 215 | 248 | 135 | 179 | 129 | 175 | F421 |
| 4 | 212 | 189 | 177 | 135 | 209 | 196 | 226 | 215 | 248 | 135 | 179 | 129 | 175 | F421 |
| 4 | 212 | 189 | 177 | 135 | 209 | 196 | 226 | 215 | 248 | 135 | 179 | 129 | 175 | F421 |
| 5 | 212 | 189 | 177 | 135 | 209 | 196 | 226 | 215 | 248 | 135 | 179 | 129 | 175 | F421 |
| 5 | 212 | 189 | 177 | 135 | 209 | 196 | 226 | 215 | 248 | 135 | 179 | 129 | 175 | F421 |
| 7 | 212 | 189 | 177 | 135 | 209 | 196 | 226 | 215 | 248 | 135 | 179 | 129 | 175 | F421 |
| 7 | 212 | 189 | 177 | 135 | 209 | 196 | 226 | 215 | 248 | 135 | 179 | 129 | 175 | F421 |
| 9 | 212 | 189 | 177 | 135 | 209 | 196 | 226 | 215 | 248 | 135 | 179 | 129 | 175 | F421 |
| 9 | 212 | 189 | 177 | 135 | 209 | 196 | 226 | 215 | 248 | 135 | 179 | 129 | 175 | F421 |
| 10 | 212 | 189 | 177 | 135 | 209 | 196 | 226 | 215 | 248 | 135 | 179 | 129 | 175 | F421 |
| 10 | 212 | 189 | 177 | 135 | 209 | 196 | 226 | 215 | 248 | 135 | 179 | 129 | 175 | F421 |
| 13 | 212 | 189 | 177 | 135 | 209 | 196 | 226 | 215 | 248 | 135 | 179 | 129 | 175 | F421 |
| 13 | 212 | 189 | 177 | 135 | 209 | 196 | 226 | 215 | 248 | 135 | 179 | 129 | 175 | F421 |
| 41 | 212 | 189 | 177 | 135 | 209 | 196 | 226 | 215 | 248 | 135 | 179 | 129 | 175 | F421 |
| 41 | 212 | 189 | 177 | 135 | 209 | 196 | 226 | 215 | 0 | 135 | 179 | 129 | 175 | Failed |
| 44 | 212 | 189 | 177 | 135 | 209 | 196 | 226 | 215 | 248 | 135 | 179 | 129 | 175 | F421 |
| 44 | 212 | 189 | 177 | 135 | 209 | 196 | 226 | 215 | 248 | 135 | 179 | 129 | 175 | F421 |
| 46 | 214 | 186 | 177 | 135 | 209 | 196 | 226 | 215 | 248 | 135 | 179 | 129 | 175 | F422 |
| 46 | 214 | 186 | 177 | 135 | 209 | 196 | 226 | 215 | 248 | 135 | 179 | 129 | 175 | F422 |
| 47 | 212 | 189 | 177 | 135 | 209 | 196 | 226 | 215 | 248 | 135 | 179 | 129 | 175 | F421 |
| 47 | 212 | 189 | 177 | 135 | 209 | 196 | 226 | 215 | 248 | 135 | 179 | 129 | 175 | F421 |
| 50 | 210 | 189 | 177 | 135 | 209 | 196 | 226 | 215 | 248 | 135 | 183 | 132 | 175 | F423 |
| 50 | 210 | 189 | 177 | 135 | 209 | 196 | 226 | 215 | 248 | 135 | 183 | 132 | 175 | F423 |
| 57 | 212 | 189 | 174 | 135 | 209 | 196 | 226 | 215 | 248 | 135 | 183 | 129 | 175 | F424 |
| 57 | 212 | 189 | 174 | 135 | 209 | 196 | 226 | 215 | 248 | 135 | 183 | 129 | 175 | F424 |
| 58 | 212 | 189 | 177 | 135 | 209 | 196 | 226 | 215 | 248 | 135 | 179 | 129 | 175 | F421 |
| 58 | 212 | 189 | 177 | 135 | 209 | 196 | 226 | 215 | 248 | 135 | 179 | 129 | 175 | F421 |
