# Supplementary material for: Bleaching causes loss of disease resistance within the threatened coral species Acropora cervicornis
Source: eLife. 2018 Sep 11;7:e35066. doi: 10.7554/eLife.35066 (PMC6133546; doi:10.7554/eLife.35066)
Supplement: Supplementary file 3. — Bold genotypes represent those that showed a significant increased risk after exposure to disease. [file elife-35066-supp3.docx]

Supplementary file 3. Results of the Bayesian relative risk analysis on the log scale when disease prevalence of corals exposed to the disease homogenate were compared with those that were exposed to the healthy homogenate prior to bleaching. Bold genotypes represent those that showed a significant increased risk after exposure to disease.

| Genotype | Lower credible interval | Median | Upper credible interval |
| --- | --- | --- | --- |
| 1 | 0.02587 | 0.8815 | 31.36 |
| 3 | 0.02453 | 0.8831 | 30.18 |
| 4 | 0.4734 | 3.374 | 97.87 |
| 5 | 0.1936 | 2.119 | 63.09 |
| 7 | 0.4681 | 3.3 | 90.97 |
| **9** | **1.085** | **5.716** | **161.2** |
| **10** | **1.348** | **6.374** | **158** |
| 13 | 0.4678 | 3.302 | 84.8 |
| 41 | 0.02842 | 0.9916 | 33.11 |
| 44 | 0.03016 | 1 | 37.2 |
| 46 | 0.9364 | 2.885 | 18.3 |
| 47 | 0.2435 | 2.35 | 64.42 |
| 50 | 0.2413 | 2.384 | 66.76 |
| 57 | 0.2342 | 2.314 | 65.11 |
| 58 | 0.5656 | 3.697 | 102.4 |
