# Supplementary material for: Bleaching causes loss of disease resistance within the threatened coral species Acropora cervicornis
Source: eLife. 2018 Sep 11;7:e35066. doi: 10.7554/eLife.35066 (PMC6133546; doi:10.7554/eLife.35066)
Supplement: Supplementary file 5. — After corals were collected they were maintained and propagated within Mote Marine Laboratory's offshore in situ coral nursery. [file elife-35066-supp5.docx]

Supplementary file 5. Collection information including date of collection, habitat type of collection site, and collection location in latitude and longitude of each coral genotype used within the present study. After corals were collected they were maintained and propagated within Mote Marine Laboratories offshore *in situ* coral nursery.

| **Genotype ID #** | **Collection Date** | **Habitat** | **Latitude** | **Longitude** |
| --- | --- | --- | --- | --- |
| **1** | 2/23/08 | Reef Margin | 24.56937 | -81.33028 |
| **3** | 2/23/08 | Offshore | 24.56307 | -81.40100 |
| **4** | 2/23/08 | Offshore | 24.56935 | -81.38168 |
| **5** | 2/22/08 | Reef Margin | 24.52257 | -81.51978 |
| **7** | 2/23/08 | Midchannel | 24.56038 | -81.50137 |
| **9** | 2/23/08 | Nearshore | 24.61440 | -81.37895 |
| **10** | 2/23/08 | Nearshore | 24.61515 | -81.37917 |
| **13** | 12/1/09 | MidChannel | 24.59592 | -81.37162 |
| **41** | 5/10/10 | Offshore | 24.55320 | -81.43758 |
| **44** | 5/21/10 | Reef Margin | 24.56860 | -81.32650 |
| **46** | 5/25/10 | midchannel | 24.56080 | -81.50143 |
| **47** | 5/25/10 | midchannel | 24.54835 | -81.53320 |
| **50** | 7/30/10 | midchannel | 24.54854 | -81.53308 |
| **57** | 11/11/10 | Offshore | 24.55948 | -81.41322 |
| **58** | 11/16/10 | Reef Margin | 24.52328 | -81.53572 |
