# Supplementary material for: Bleaching causes loss of disease resistance within the threatened coral species Acropora cervicornis
Source: eLife. 2018 Sep 11;7:e35066. doi: 10.7554/eLife.35066 (PMC6133546; doi:10.7554/eLife.35066)
Supplement: Supplementary file 6. [file elife-35066-supp6.docx]

Supplementary file 6. Experimental design and results of the four different trials used to quantify relative risk of disease for 15 different genotypes of *Acropora cervicornis*

|  | **Trial 1** | | | |
| --- | --- | --- | --- | --- |
| Genotype | # of disease  exposed ramets | # of control  ramets | # of diseased  exposed that died | # of controls  that died |
| 1 | 1 | 1 | 0 | 0 |
| 3 | 1 | 1 | 0 | 0 |
| 4 | 1 | 1 | 1 | 0 |
| 5 | 1 | 1 | 0 | 0 |
| 7 | 1 | 1 | 1 | 0 |
| 9 | 1 | 1 | 1 | 0 |
| 10 | 1 | 1 | 0 | 0 |
| 13 | 1 | 1 | 0 | 0 |
| 41 | 0 | 0 | na | na |
| 44 | 0 | 0 | na | na |
| 46 | 1 | 1 | 1 | 0 |
| 47 | 0 | 0 | na | na |
| 50 | 0 | 0 | na | na |
| 57 | 0 | 0 | na | na |
| 58 | 0 | 0 | na | na |
|  |  |  |  |  |
|  | **Trial 2** | | | |
| Genotype | # of disease  exposed ramets | # of control ramets | # of diseased  exposed that died | # of controls  that died |
| 1 | 4 | 3 | 0 | 0 |
| 3 | 4 | 3 | 0 | 0 |
| 4 | 4 | 3 | 0 | 0 |
| 5 | 4 | 3 | 1 | 0 |
| 7 | 4 | 3 | 0 | 0 |
| 9 | 4 | 3 | 2 | 0 |
| 10 | 0 | 0 | na | na |
| 13 | 4 | 3 | 0 | 0 |
| 41 | 0 | 0 | na | na |
| 44 | 0 | 0 | na | na |
| 46 | 4 | 3 | 2 | 1 |
| 47 | 0 | 0 | na | na |
| 50 | 0 | 0 | na | na |
| 57 | 0 | 0 | na | na |
| 58 | 0 | 0 | na | na |
|  |  |  |  |  |
|  | **Trial 3** | | | |
| Genotype | # of disease  exposed ramets | # of control ramets | # of diseased  exposed that died | # of controls that died |
| 1 | 2 | 2 | 0 | 0 |
| 3 | 2 | 2 | 0 | 0 |
| 4 | 2 | 2 | 1 | 0 |
| 5 | 2 | 2 | 1 | 0 |
| 7 | 2 | 2 | 1 | 0 |
| 9 | 2 | 2 | 2 | 0 |
| 10 | 2 | 2 | 4 | 0 |
| 13 | 2 | 2 | 2 | 0 |
| 41 | 5 | 5 | 0 | 0 |
| 44 | 5 | 5 | 0 | 0 |
| 46 | 2 | 2 | 2 | 0 |
| 47 | 5 | 5 | 1 | 0 |
| 50 | 5 | 5 | 1 | 0 |
| 57 | 5 | 5 | 1 | 0 |
| 58 | 5 | 5 | 2 | 0 |
|  |  |  |  |  |
|  | **Trial 4** | | | |
| Genotype | # of disease  exposed ramets | # of control ramets | # of diseased  exposed that died | # of controls  that died |
| 1 | 5 | 5 | 4 | 1 |
| 3 | 5 | 5 | 0 | 0 |
| 4 | 5 | 5 | 5 | 2 |
| 5 | 5 | 5 | 5 | 1 |
| 7 | 5 | 5 | 0 | 0 |
| 9 | 5 | 5 | 5 | 1 |
| 10 | 5 | 5 | 4 | 1 |
| 13 | 5 | 5 | 4 | 2 |
| 41 | 5 | 5 | 4 | 0 |
| 44 | 5 | 5 | 4 | 0 |
| 46 | 5 | 5 | 5 | 0 |
| 47 | 5 | 5 | 5 | 2 |
| 50 | 5 | 5 | 4 | 0 |
| 57 | 5 | 5 | 4 | 2 |
| 58 | 5 | 5 | 2 | 1 |
