# Supplementary material for: Bleaching causes loss of disease resistance within the threatened coral species Acropora cervicornis
Source: eLife. 2018 Sep 11;7:e35066. doi: 10.7554/eLife.35066 (PMC6133546; doi:10.7554/eLife.35066)
Supplement: Supplementary file 7: [file elife-35066-supp7.docx]

Supplementary file 7: OpenBUGS Code for Relative Risk Analysis

####Relative Risk and Odds Ratio Disease Model

#Where “experimental” refers to corals exposed to disease homogenate

#Where “control” refers to corals exposed to healthy homogenate

#RRISK refers to relative risk

#OR refers to odds ratio

# Where “a” is the proportion of experimental corals with disease

# Where “b” is the proportion of experimental corals with no disease

# Where “c” is the proportion of control corals with disease

# Where “d” is the proportion of control corals without disease

# Where “h” is the total number of experimental corals with disease

# Where “r” is the total number of experimental corals

# Where “l” is the total number of control corals with disease

# Where “m” is the total number of control corals

model

{

for (i in 1:k) {

#Prior

a[i] ~dbeta(1,1)

c[i] ~dbeta(1,1)

# Likelihood

h[i] ~ dbin(a[i],r[i])

l[i] ~ dbin(c[i],m[i])

RRISK[i] <- a[i]/c[i]

OR[i]<- (a[i]/(1-a[i]))/(c[i]/(1-c[i]))

lnOR[i]<-log(OR[i])

ppos[i]<-step(OR[i]-1)

}

}

##August trials

Data list(k=15,

h = c(0,0,2,1,2,4,4,2,0,0,5,1,1,1,2),

r=c(7,7,7,7,7,7,5,7,5,5,7,5,5,5,5),

l = c(0,0,0,0,0,0,0,0,0,0,1,0,0,0,0),

m=c(6,6,6,6,6,6,5,6,5,5,6,5,5,5,5))

###September trial

Data list(k=15,

h = c(4,0,5,5,0,5,4,4,4,4,5,5,4,4,2),

r=c(5,5,5,5,5,5,5,5,5,5,5,5,5,5,5),

l = c(1,0,2,1,0,1,1,2,0,0,0,2,0,2,1),

m=c(5,5,5,5,5,5,5,5,5,5,5,5,5,5,5))
