# Supplementary material for: Bleaching causes loss of disease resistance within the threatened coral species Acropora cervicornis
Source: eLife. 2018 Sep 11;7:e35066. doi: 10.7554/eLife.35066 (PMC6133546; doi:10.7554/eLife.35066)
Supplement: Supplementary file 8. [file elife-35066-supp8.docx]

Supplementary file 8. *Acropora cervicornis* multilocus genotypes using 4 previously published microsatellite markers (Baums et al. 2005).

| **Field ID** | **166-1** | **166-2** | **181-1** | **181-2** | **182-1** | **182-2** | **207-1** | **207-2** | **Genotype ID** |
| --- | --- | --- | --- | --- | --- | --- | --- | --- | --- |
| **1** | 140 | 143 | 156 | 177 | 161 | 170 | 158 | 158 | C1153 |
| **3** | 140 | 146 | 174 | 183 | 161 | 167 | 164 | 167 | C1155 |
| **4** | 143 | 146 | 174 | 177 | 173 | 188 | 158 | 158 | C1156 |
| **5** | 143 | 146 | 171 | 174 | 161 | 161 | 158 | 167 | C1157 |
| **7** | 140 | 143 | 171 | 177 | 161 | 164 | 158 | 158 | C1159 |
| **9** | 140 | 140 | 156 | 168 | 161 | 167 | 158 | 164 | C1161 |
| **10** | 146 | 146 | 162 | 162 | 161 | 170 | 158 | 158 | C1083 |
| **13** | 140 | 149 | 168 | 171 | 143 | 167 | 158 | 161 | C1365 |
| **41** | 140 | 149 | 174 | 174 | 161 | 167 | 158 | 158 | C1374 |
| **44** | 140 | 143 | 162 | 165 | 161 | 161 | 158 | 164 | C1459 |
| **46** | 140 | 140 | 162 | 171 | 155 | 167 | 158 | 158 | C1507 |
| **47** | 137 | 140 | 171 | 174 | 137 | 158 | 158 | 158 | C1376 |
| **50** | 140 | 146 | 171 | 177 | 161 | 167 | 158 | 158 | C1170 |
| **57** | 140 | 143 | 168 | 171 | 155 | 158 | 158 | 173 | C1511 |
| **58** | 143 | 146 | 174 | 177 | 158 | 185 | 158 | 158 | C1462 |
