# Supplementary material for: Bleaching causes loss of disease resistance within the threatened coral species Acropora cervicornis
Source: eLife. 2018 Sep 11;7:e35066. doi: 10.7554/eLife.35066 (PMC6133546; doi:10.7554/eLife.35066)
Supplement: Supplementary file 9. [file elife-35066-supp9.docx]

Supplementary file 9. Standardized grouping information to identify each operational taxonomic unit to the most accurate taxonomic level.

| **Identity to reference sequence** | **Identity Designation** |
| --- | --- |
| > 97% | Species |
| Between 97% and 95% | (unclassified Genus) |
| Between 95% and 90% | (unclassified Family) |
| Between 90% and 85% | (unclassified order) |
| Between 85% and 80% | (unclassified class) |
| Between 80% and 77% | (unclassified phylum) |
| < 77% | (unknown) |
